# The Role of Teachers' Expectations in the Association between Children's SES and Performance in Kindergarten: A Moderated Mediation Analysis

**DOI:** 10.1371/journal.pone.0034502

**Published:** 2012-04-10

**Authors:** Sara Speybroeck, Sofie Kuppens, Jan Van Damme, Peter Van Petegem, Carl Lamote, Tinneke Boonen, Jerissa de Bilde

**Affiliations:** 1 Education and Training Research Group, Department of Educational Sciences, K. U. Leuven, Leuven, Belgium; 2 Methodology of Educational Sciences Research Group, Department of Educational Sciences, K. U. Leuven, Leuven, Belgium; 3 EMGO Institute for Health and Care Research, VU University Amsterdam, Amsterdam, The Netherlands; 4 Research Group EduBROn, Institute of Education and Information Sciences, University of Antwerp, Antwerp, Belgium; University of Minnesota, United States of America

## Abstract

This study examines the role of teachers' expectations in the association between children's socio-economic background and achievement outcomes. Furthermore, the role of children's ethnicity in moderating this mediated relation is investigated. In the present study, 3,948 children from kindergarten are examined. Data are analysed by means of structural equation modeling. First, results show that teachers' expectations mediate the relation between children's SES and their later language and math achievement, after controlling for children's ethnicity, prior achievement and gender. This result indicates that teachers may exacerbate individual differences between children. Second, children's ethnicity moderates the mediation effect of teachers' expectations with respect to math outcomes. The role of teachers' expectations in mediating the relation between SES and math outcomes is stronger for majority children than for minority children.

## Introduction

While interacting with students, teachers develop expectations for students' academic performance and social skills [1]. The influence of these expectations has been the focus of debate for many decades [2]. Rosenthal and Jacobson [3] were the first to study the effect of expectations on students' academic progress in a low-income elementary school. They concluded that students whose teachers expected a high increase of learning ability within the next year, indeed had higher intelligence scores at the end of the school year. Although the validity of the study was criticized [4], [5], the Pygmalion study led to an increasing interest in examining the effects of teachers' expectations in the classroom.

### Predictors of Teachers' Expectations

Research has shown that teachers base their expectations on both objective (e.g., students' past achievement) and subjective (e.g., teachers' prejudices) information [6]. Students' early performance and gender predicted teachers' expectations, with higher expectations for high achieving students and girls [7], [8]. According to some authors, expectations can also be based on students' social class and ethnicity [1], [8], [9], [10], [11]. In general, studies show that teachers have lower expectations for minority students and students with a lower socio-economic status (SES) than for majority students and students with a higher SES. However, other studies failed to find effects of social class and ethnicity [7], [10]. According to Madon et al. [7], teachers are more likely to base their expectations on students' achievement and motivation. Thus, results with respect to SES and ethnicity as predictors of teachers' expectations have been inconsistent. Furthermore, most studies have investigated the role of either social class or ethnicity separately. This is problematic because these phenomena are closely related. On average, minority children have a lower SES than majority children [11]. In a study of Rubie-Davies, Hattie, and Hamilton [12], both social class and ethnicity were included as predictors of teachers' expectations. In contrast to what was hypothesized, the authors found that teachers' expectations differed for students by ethnicity rather than by social class. Because currently no consensus exists about the way students' social class and ethnicity influence teachers, it would be interesting to examine further whether and how these child characteristics interact in predicting the teachers' expectations.

### Effects of Teachers' Expectations

Not only do teachers form expectations regarding their students, these expectations may also relate to student outcomes. They can affect the teacher-student interactions in a manner that leads the student to fulfil the teachers' expectations [13]. A range of studies have demonstrated an effect of teachers' expectations in various subject areas, such as mathematics [8], [14], [15], [16], [17], reading [8], [17], [18], [12] and sport education [6], [13]. However, the effect reported in the literature is rather small, ranging from *r* = .10 to .20 [2].

Because of these small effects, the focus in research has no longer been on the effect itself, but rather on identifying when and for whom it occurs [13]. In some situations, or for some people, the effects of expectations on students' outcomes are stronger. Jussim and Harber [2] for instance, reported stronger effects early in the school year, when teachers were not yet familiar with their students. McKown and Weinstein [19] investigated the role of ethnicity as a potential moderator of the relation between teachers' expectations and students' math and reading achievement. The authors found that children from academically stigmatized groups (i.e., African American children) were more susceptible to the effect than the non stigmatized groups (i.e., Caucasian children). In the reading domain, Hinnant et al. [8] came to similar results. They found that teachers' expectations were more strongly related to later performance for minorities (non-White). In contrast, others failed to find a moderation effect of ethnicity [10], [20]. In these studies, teachers' expectations did not interact with students' ethnicity in predicting students' performance.

### An Integrated Model of Teachers' Expectations

In this paper we propose a structural equation modeling approach to examine whether teachers base their expectations on children's socio-economic background and whether these expectations in turn affect children's language and math outcomes. Furthermore, the role of children's ethnicity in moderating these effects was investigated.

First, an integrated mediation model was tested in which teachers' expectations mediate the association between children's SES and outcomes after controlling for children's ethnicity, gender and previous language and math achievement scores. These control variables were chosen based on their relevance in predicting later children's achievement outcomes. In accordance with previous studies [9], we assumed that teachers would have higher expectations for children with a higher SES and lower expectations for children with a lower SES. Furthermore, teachers' expectations can affect later children's outcomes because of a differential treatment in the class [11]. Therefore, we expected that these higher/lower expectations (based on children's higher/lower socio-economic background) in turn would lead to higher/lower achievement outcomes in language and math. In that way, teachers may exacerbate the achievement gap of children from different socio-economic backgrounds [20].

Second, we examined whether the integrated mediation model would differ across majority and minority children. A moderated mediation model was tested, in which the strength of an indirect effect varies across the levels of the moderator [21]. As mentioned earlier, ethnicity plays a role both in the origination of teachers' expectations as in the effect of these expectations on later students' performance [11], [19]. Given these results, it seems likely that ethnicity would also moderate the indirect effect of SES on children's outcomes through teachers' expectations. Because the presence of multiple vulnerabilities strengthened the direct associations in previous research, we assumed that the indirect association would also be stronger for minority than for majority children.

## Methods

### Ethics Statement

The data were anonymous, using publicly available secondary data.

### Data

The data were collected in the context of the large-scale longitudinal SiBO-project (i.e., the Dutch acronym for School Careers in Elementary Education) [22]. The project initiated in 2002 and intended to describe and explain inter-individual differences in children's developmental trajectories throughout elementary school in Flanders (Dutch speaking part of Belgium). For that purpose, a cohort of approximately 4,000 students was followed from kindergarten (age 5–6) until the end of sixth grade (age 11–12) and beyond. The SiBO project involved a random stratified sample of 122 schools. Stratification was based on educational network and school size. The sample was representative for the entire Flemish school population in terms of the applied stratification criteria, the geographic area and the proportion of disadvantaged students targeted by the Act of Equal Opportunities in Education [23]. In these sampled schools, 3,949 children attended kindergarten during the school year 2002–2003. This group of children comprised the sample for the present study. At the start of that school year, children's average age was 5 years and 10 months. In Flanders, attending kindergarten is voluntary. Nevertheless, 99% of all the 4–5 year olds are going to kindergarten on a regularly basis [24].

### Instruments

All the instruments that were used to operationalize the variables yielded adequate internal consistency, with Cronbach's alpha ranging from .83 to .93.

#### Standardized Language and Math Achievement Test

Language and math achievement was assessed at the beginning (September 2002) and in the end (May 2003) of the school year [25]. The language achievement test consisted of 40 items divided into five subtests: listening comprehension, sound and rhyme, auditory sequencing, literacy knowledge, and sound blending [26]. The math achievement test assessed skills in number sense such as comparing magnitudes, counting, and understanding mathematical concepts [27].

#### Students' Characteristics

The students' characteristics gender, SES and ethnicity were based on data from a parent questionnaire administered in February 2003 [28]. The variable *gender* was represented by a dichotomous variable with a score of “1” for boys (50.8%) and “0” for girls (49.2%). The latent construct *SES* represents the socio-economic status of the child's family and was composed of five items: the educational level of mother and father (rated on a 5-point Likert scale), the occupation of mother and father (rated on a 7-point Likert scale) and the monthly household income (rated on a 6-point Likert scale). Parents' nationality at birth was used as an indicator of *ethnicity*. Children were classified into one of 2 categories: both parents had Belgian nationality at birth (majority children 80.2%, coded as 0) and one of the parents or both parents had a foreign nationality (minority children 19.8%, coded as 1).

#### Teachers' Expectations

Teacher questionnaires were used to assess the teachers' expectations [29]. In the middle of the academic year (February 2003), teachers were given a questionnaire in which they had to answer questions concerning each child in their classroom. The latent construct teachers' expectations in the current study covers 4 items: “the child is highly gifted” (rated on a 6-point Likert scale), “the child will need extra care in the future” (rated on a 6-point Likert scale and inversed), “the child will be able to succeed in higher education” (rated on a 4-point Likert scale) and “the child is performing better relative to his or her peers” (rated on a 3-point Likert scale). These items were chosen based on their correspondence to the items generally used in teacher expectation research. Dusek and Joseph [1] defined academic expectations as teachers' perceptions of students' performance, achievement, ability and attainment. The first item covering teachers' expectations in our study (i.e., “the child is highly gifted”) corresponds to one of the items used by Jussim and Eccles [15] (i.e., “how much natural mathematical talent does this student have?”) and to two of the items used by Van den Bergh et al. [20] (i.e., “he or she is a smart student” and “he or she is an intelligent student”). The second and third item covering teachers' expectations in the current study (i.e., “the child will need extra care in the future” and “the child will be able to succeed in higher education”) provide a measure for teachers' perceptions of students' future performance and attainment. A comparable item is “he or she will probably have a successful school career” used by Van den Bergh et al. [20] to measure expectations. The fourth item (i.e., “the child is performing better relative to his or her peers”) corresponds to another item used by Jussim and Eccles [15] to assess teachers' expectations (i.e., “compared to other students in this class, how well is this student performing in math?”). The internal consistency of our expectation scale was found to be good (Cronbach's α = .83).

### Data Analysis Strategy

Data were analysed by means of Structural Equation Modeling (SEM). SEM is a data analysis method incorporating many other traditional analysis techniques [30]. In SEM, complex models can be fitted, which involve a number of linear equations and in which measurement error is allowed in the dependent and the independent variables [31]. Furthermore, it is possible to specify latent variable models that provide separate estimates of relations between the latent variables and their indicators (measurement model) and of the relations among the latent constructs (the structural model) [32]. Another advantage described by Tomarken and Waller [32] is that the fit of different alternative models can be evaluated comparatively. To account for the hierarchical structure of the data during analyses, the classroom identification number of each child was used as a cluster variable.

Parameter estimates and goodness-of-fit indices were computed using Mplus (Version 3.0 [33]) in combination with STREAMS (Version 3.0 [34]). Because our data contained missing values and deviated from normality at the univariate and multivariate level, a robust maximum likelihood estimator (MLR) was used, with the asymptotic covariance matrix as input. MLR estimations result in model parameter estimates and standard errors that are robust to missing data and violations of normality [35]. To evaluate the size of the effects, Cohen's effect size index *f^2^* was used with *f^2^* = 0.35 indicating a large effect, *f^2^* = 0.15 a medium effect and *f^2^* = 0.02 a small effect [36].

The models' goodness-of-fit were evaluated using a modified chi-squared statistic that is based on the Yuan-Bentler T2* test statistic (Y-Bχ^2^) [35]. Additional goodness-of-fit measures, such as the Root Mean Square Error of Approximation (RMSEA), the Standardized Root Mean Square Residual (SRMR) and the Comparative Fit Index (CFI), were used to evaluate the models since the χ^2^ measure is sensitive to sample size. According to Schermelleh-Engel, Moosbrugger, and Müller [37], RMSEA and SRMR values of .05 or lower, and CFI values of .97 or higher indicate a good fit, while RMSEA and SRMR values between .05 and .08 and CFI values between .95 and .97 indicate an acceptable fit. The Satorra-Bentler-Scaled-χ^2^-difference-test (ΔSBS-χ^2^) was used to compare nested models.

**Figure 1:**
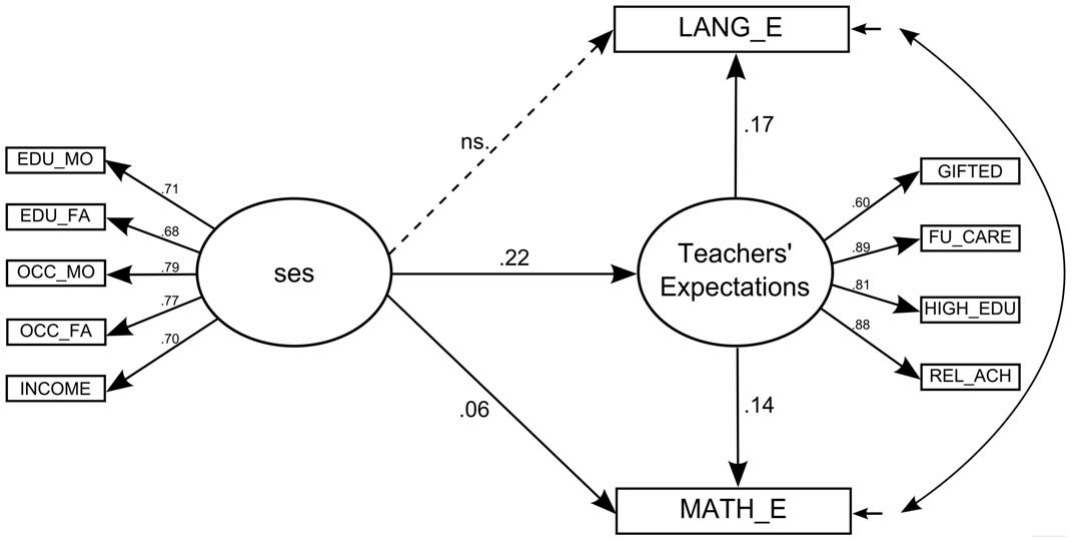
Mediation model of teachers' expectations. In this model, the residuals of the language and math achievement outcomes are allowed to correlate. For reasons of clarity, paths from the control variables (ethnicity, gender and prior language and math achievement) to later achievement are not shown. All coefficients are standardized. Except for the path ses-LANG_E, all coefficients are significant (*p*<.001). EDU_MO = education of the mother; EDU_FA = education of the father; OCC_MO = occupation of the mother; OCC_FA = occupation of the father; FU_CARE = future care; HIGH_EDU = higher education; REL_ACH = relative achievement; LANG_E = language achievement at the end of the school year; MATH_E = math achievement at the end of the school year.

The proposed mediation model, in which children's SES is related to children's language and math outcomes, was tested following different steps (cf. Holmbeck [38]). First, a model was fitted in which SES was related to children's outcomes after controlling for children's ethnicity, gender and prior language and math achievement (direct effect model). Second, the latent construct ‘teachers’ expectations' was included in the model (full model, see Figure 1). To test the mediation effect of teachers' expectations between children's SES and their later outcomes, the full model was compared to a model in which the direct path between SES and later achievement was constrained to zero. When these two models do not differ significantly from each other and when the initial significant relation between SES and outcomes is reduced to non-significant after the mediator is included, full mediation is shown. When a full mediation model is not confirmed, partial mediation can still be present [39]. Partial mediation is demonstrated when the direct effect is reduced, but still different from zero after the inclusion of the mediator [40].

To examine whether the structural paths and mediation effects were different for majority and minority children, multi-group modeling was used. With this technique the fit of a constrained model, in which all the structural coefficients of the model are set equal across groups, is compared with the fit of a more unconstrained model, in which some of the coefficients are allowed to vary across the groups. A significant scaled-χ^2^-difference-test implies that there is a significant difference between the two groups [41]. To test whether the indirect effect differs between majority and minority children (moderated mediation), a few procedures have been recommended in the literature [42]. One of the approaches involves adding a nonlinear constraint in the model. This means that the two different mediation effects that are being compared, are constraint to be equal across the groups. The constrained and unconstrained models are then compared using the Wald chi-square test. This test is conducted by dividing the product of the direct effects (i.e., the indirect effect) by its standard error and comparing the result to a standard normal distribution [43]. A statistically significant Wald test indicates that the indirect effects are significantly different between the two groups. It should be noted that this approach is equivalent to the subgroup approach described by Edwards [44]. However, in contrast to most studies in which the structural paths are analysed separately, we analyse moderated mediation by using the product term of the different mediation paths.

## Results

### Descriptive Statistics and Correlations

The descriptive statistics are presented in Table 1. Correlations among the indicators are, with the exception of several gender correlations, all significant and mostly positive (see Table 2).

**Table 1:** Descriptive Statistics.

Measure	Min.	Max.	M	SD	%	% missing
LANG_B	13.89	69.56	44.42	10.36		4.3
MATH_B	16.27	61.44	43.22	9.57		5.1
LANG_E	17.52	71.80	52.06	9.31		4. 5
MATH_E	25.54	67.98	51.56	8.88		5.1
EDU_MO	1	7	4.44	1.77		11.6
EDU_FA	1	7	4.47	1.65		12.9
OCC_MO	1	5	3.24	1.02		11.6
OCC_FA	1	5	3.28	1.05		15.6
INCOME	1	6	3.01	1.14		26.3
GIFTED	1	6	2.35	1.24		5.1
FU_CARE	1	6	3.93	1.55		4.3
HIGH_EDU	1	4	2.78	0.91		10.3
REL_ACH	1	3	2.24	0.77		6.1
GENDER (boy)					50.80	0.03
ETHNICITY (minority)					19.80	14.2

*Note.* LANG_B = language achievement at the beginning of the school year; MATH_B = math achievement at the beginning of the school year; EDU_MO = education of the mother; EDU_FA = education of the father; OCC_MO = occupation of the mother; OCC_FA = occupation of the father; FU_CARE = future care; HIGH_EDU = higher education; REL_ACH = relative achievement; LANG_E = language achievement at the end of the school year; MATH_E = math achievement at the end of the school year.

**Table 2:** Correlations.

		1	2	3	4	5	6	7	8	9	10	11	12	13	14
1	LANG_B														
2	MATH_B	.76**													
3	GENDER	−.07**	−.01												
4	ETHNICITY	−.30**	−.33**	−.02											
5	EDU_MO	.25**	.31**	.01	−.14**										
6	EDU_FA	.30**	.34**	.01	−.24**	.46**									
7	OCC_MO	.31**	.38**	.01	−.23**	.47**	.58**								
8	OCC_FA	.25**	.29**	.04*	−.12**	.61**	.40**	.62**							
9	INCOME	.29**	.35**	.03	−.25**	.54**	.48**	.52**	.49**						
10	GIFTED	.39**	.44**	−.01	−.12**	.23**	.21**	.25**	.24**	.25**					
11	FU_CARE	.55**	.59**	−.11**	−.15**	.26**	.27**	.30**	.25**	.27**	.46**				
12	HIGH_EDU	.57**	.62**	−.07**	−.16**	.37**	.37**	.41**	.36**	.38**	.56**	.71**			
13	REL_ACH	.57**	.61**	−.07**	−.11**	.27**	.28**	.31**	.27**	.27**	.53**	.73**	.79**		
14	LANG_E	.71**	.70**	−.08**	−.24**	.23**	.28**	.30**	.23**	.28**	.37**	.52**	.57**	.54**	
15	MATH_E	.71**	.85**	.02	−.33**	.30**	.35**	.39**	.31**	.37**	.42**	.58**	.62**	.60**	.74**

*Note.* LANG_B = language achievement at the beginning of the school year; MATH_B = math achievement at the beginning of the school year; EDU_MO = education of the mother; EDU_FA = education of the father; OCC_MO = occupation of the mother; OCC_FA = occupation of the father; FU_CARE = future care; HIGH_EDU = higher education; REL_ACH = relative achievement; LANG_E = language achievement at the end of the school year; MATH_E = math achievement at the end of the school year;

** *p*<.01;

* *p*<.05.

### Measurement Model

A confirmatory factor analysis was conducted with eight latent variables and 15 indicators. The latent construct SES was indexed by 5 indicators, i.e., ‘education of the mother’, ‘education of the father’, ‘occupation of the mother’, ‘occupation of the father’ and ‘family income’. The construct teachers' expectations was covered by 4 indicators, i.e., ‘gifted’, ‘future care’, ‘higher education’ and ‘relative achievement’. Ethnicity, gender and achievement for language and math (prior and later) were each represented by a single indicator with the error variance fixed to zero. Estimation of the measurement model indicated a good fit (Y-Bχ^2^(68) = 741.68; RMSEA = .05; SRMR = .03; CFI = .97). The standardized factor loadings ranged from .60 to .89. Correlations between the latent constructs showed that SES was significantly related to teachers' expectations (*r* = .52, *p*<.001), later language achievement (*r* = .39, *p*<.001) and later math achievement (*r* = .49, *p*<.001). Furthermore, teachers' expectations was significantly related to later language achievement (*r* = .64, *p*<.001) and later math achievement (*r* = .70, *p*<.001). All other variables were significantly correlated (ranging from *r* = .07 to .85, *p*<.001), except for the correlations of gender with SES, ethnicity and prior language and math achievement.

### Mediation Models

The first, direct model, in which SES was related to children's language and math outcomes, demonstrated an acceptable fit to the data (Y-Bχ^2^ (29) = 581.64; RMSEA = .07; SRMR = .03; CFI = .96). Children's SES was significantly associated with later language (β = .04, *p*<.05, *f^2^* = .002) and math (β = .09, *p*<.001, *f^2^* = .01) outcomes, even after controlling for children's ethnicity, gender and prior achievement. It should be noted that in terms of magnitude, the effect of SES was quite small. Nevertheless, even small effects can have a large impact on children's outcomes if they accumulate over time [2], [14], [45], [46].

The full model, in which the construct ‘teachers’ expectations' was included, is presented in Figure 1. This mediation model yielded a good fit (Y-Bχ^2^ (69) = 804.66; RMSEA = .05; SRMR = .03; CFI = .96). Next, the mediation effect of teachers' expectations between SES and later outcomes was tested separately for language and math outcomes. First, for language, the full model was compared to a model in which the path from SES to language achievement was constrained to zero. This constrained model also had a good fit (Y-Bχ^2^ (70) = 799.92; RMSEA = .05; SRMR = .03; CFI = .96), but the χ^2^-value became smaller. Performing a SBS-χ^2^-difference test would thus result in a negative χ^2^ statistic. Such an outcome is possible and indicates that the two models are very close in fit [47], [48]. We therefore conclude that constraining the path between SES and language achievement does not worsen model fit. Moreover, the initially significant association between SES and language achievement (β = .04, *p*<.05, *f^2^* = .002) was reduced to non-significant (β = .00, *p* = ns. *f^2^* = .000). These results indicate that teachers' expectations fully mediated the association between children's SES and their language achievement scores. Higher SES was significantly associated (β = .22, *p*<.001, *f^2^* = .05) with higher teachers' expectations, which in turn were significantly associated (β = .17, *p*<.001, *f^2^* = .03) with higher language outcomes at the end of the school year.

Second, to test the mediation effect of teachers' expectations between SES and math achievement, the full model was also compared to a model in which the path from SES to later math achievement was constrained to zero. Constraining this path did significantly worsen model fit (ΔSBS-χ^2^ (1) = 12.84, *p*<.001). This indicates that the direct path between SES and math achievement should be included in the model, thus rejecting a full mediation effect of teachers' expectations. Nevertheless, the reduction in the size of direct association after the mediator was included in the model, pointed to partial mediation. Thus, SES was positively associated (β = .22, *p*<.001, *f^2^* = .05) with teachers' expectations, which in turn were positively associated (β = .14, *p*<.001, *f^2^* = .02) with math outcomes at the end of the school year. In addition, a higher SES was also directly associated with higher later math outcomes (β = .06, *p*<.001, *f^2^* = .004).

### Moderation Models

To examine whether children's ethnicity moderated the mediated effect of teachers' expectations between children's SES and achievement, a moderated mediation model was fitted. As mentioned above, moderated mediation was tested by comparing a model in which the mediation effect was constrained to be equal across the majority and the minority group, with a model in which the mediation effect was allowed to vary across both groups. Results indicated that for language, the fit of the fully constrained model did not significantly differ from the fit of the more unconstrained model in which the mediated effect was allowed to vary across the groups (Δχ^2^ (1) = 3.11, *p* = ns.). This indicates that children's ethnicity did not moderate the fully mediated effect of SES on language achievement through teachers' expectations. For math achievement, the fit of the fully constrained model significantly differed from the fit of the unconstrained model (Δχ^2^ (1) = 12.93, *p*<.001). This suggests that the partially mediated effect of SES on math outcomes through teachers' expectations significantly differed for majority and minority children. Looking at the standardized regression coefficients, this mediated effect is slightly stronger for majority (β = .04, *f^2^* = .002) than for minority (β = .02 *f^2^* = .0004) children.

To further examine which specific paths of the mediated effect for math differed between majority and minority children, additional multi-group analyses were performed. First, we examined whether ethnicity moderated the association between SES and teachers' expectations. Results demonstrated that the fit of the fully constrained model significantly differed from the fit of the more unconstrained model in which the path between SES and teachers' expectations was allowed to vary across groups (ΔSBS-χ^2^ (1) = 5.89, *p*<.05). This indicates that there was a difference between majority and minority children concerning the association between SES and teachers' expectations, with a stronger association for majority children (β = .24, *f^2^* = .06) than for minority children (β = .14, *f^2^* = .02). Second, the fully constrained model was also compared to a model in which the path between teachers' expectations and math outcomes was allowed to vary across groups. Results showed significantly different fits between both models (ΔSBS-χ^2^ (1) = 20.62, *p*<.001). The association between teachers' expectations and math achievement was different for majority and minority children. The effect of teachers' expectations seemed to be somewhat stronger for majority (β = .16, *f^2^* = .03) than for minority children (β = .11, *f^2^* = .01).

## Discussion

The purpose of this study was to test an integrated mediation model in which children's socio-economic background is associated with their later language and math outcomes through teachers' expectations. Furthermore, the role of ethnicity in moderating these associations was examined. First, findings revealed that teachers' expectations mediate the relation between children's SES and later language and math achievement, after controlling for children's ethnicity, prior achievement and gender. Second, children's ethnicity moderated the mediation effect of teachers' expectations with respect to math outcomes. These findings will be discussed in more detail in the following sections.

### Socio-Economic Background and Teachers' Expectations

Research has indicated that children's socio-economic background plays a significant role in predicting teachers' beliefs and expectations. In general, teachers have lower expectations for children from lower socio-economic backgrounds [1]. Results of the current study confirmed these findings. Kindergarten teachers in our study judged children with higher SES as more favourably than children of lower socio-economic backgrounds over and above children's ethnicity, gender and prior achievement (β = .22, *f^2^* = .05). Furthermore, the current study extended previous research by investigating interaction effects of children's SES with ethnicity in predicting teachers' expectations. In contrast to what we expected, the association between SES and expectations was stronger for majority children than for minority children. Results showed that for majority children, teachers were more inclined to base their expectations on the SES of the child. For minority children, teachers made less distinction between high and low SES levels. A possible explanation for this result may be that most kindergarten teachers in our sample are from the ethnic majority group themselves [49] and consequently they pay less attention to (more subtle) SES differences of minority children than to SES differences of children from the same ethnic background.

### Teachers' Expectations and Achievement

Teachers' expectations in our study predicted children's language and math achievement at the end of the school year. This result concurs with previous studies concerning teacher expectation effects [15], [17], [8]. However, the size of the effects (*f^2^* = .03 for language *f^2^* = .02 for math) is smaller than the average effect of *r* = .10 to .20 found in prior research [2]. Therefore, it is important to interpret the results accordingly and not to overestimate their significance. Nevertheless, as mentioned previously, several authors have argued that even small effects can have a large impact on children's outcomes if these effects accumulate over time [2], [14], [45], [46].

When looking at the difference between majority and minority children for the effect of teachers' expectations on children's math outcomes, a moderation effect was found. However, in contrast to our hypothesis, the effect was larger for majority than for minority children. Majority children seem to be more susceptible to the teachers' expectations than minority children. This could indicate that the expectations, based on children's socio-economic background, are of lesser importance for minority children than originally thought [12]. A positive consequence for these children is that lower teachers' expectations do not automatically lead to lower performance. On the other hand, minority children can take less advantage of the positive consequences of higher teachers' expectations. Again, this result can be explained by the fact that most teachers in Flanders belong to the ethnic majority group. Possibly, the teachers are better at engaging in stimulating interactions with majority children [51]. They might be better at encouraging and praising children with a similar ethnic background as their own.

### An Integrated Model

Results of our integrated model showed that teachers' expectations fully mediated the association between children's SES and language outcomes and partially mediated the association between SES and math outcomes. These results suggest that teachers base their expectations on children's SES and these expectations in turn affect children's later outcomes. Teachers have lower/higher expectations for children from lower/higher socio-economic backgrounds and these expectations lead to lower/higher achievement, even after controlling for children's prior achievement, gender and ethnicity. Thus, the expectations of teachers can be seen as one of the possible links between children's SES and their outcomes. That is, the effect of socio-economic background on achievement is at least in part due to teachers' expectations. Teachers may play a role in exacerbating existing individual differences between children [45]. This finding is an illustration of the “Matthew effect” [52]. The effect refers to a process of cumulative advantage or disadvantage following initial advantage or disadvantage [53]. Children with a lower socio-economic background arrive at kindergarten with lower levels of competence due to family circumstances [54]. In turn, these children are less rewarded for their knowledge [55] and more impeded by their teachers [56] than children with a higher socio-economic background. As a result, lower socio-economic background children tend to fall even further behind over time [54].

Another finding of this study is that children's ethnicity moderated the mediation relation of teachers' expectations between children's SES and math outcomes. In contrast to what we expected, the effect was slightly stronger for majority than for minority children. It thus seems that children from minority and majority backgrounds are affected differently by the expectations of teachers that exacerbate the actual individual SES differences between children in kindergarten.

### Limitations and Future Research

The results in our study need to be understood in the context of some limitations.

First, it should be noted that the cross-sectional design of the study does not allow to draw causal inferences. In the current study, teachers' expectations functioned as a predictor of children's outcomes. However, the relation between teacher and student likely reflects rather a bidirectional than a unidirectional process [50]. It is not only the teacher who influences the student or the student who influences the teacher, teacher and students mutually influence each other. In order to address these findings, cross-lagged panel research is needed.

Second, it is always possible that other factors that were not assessed in the current study, may affect predictors and outcomes (omitted variable problem). This study has focused on student characteristics predicting teachers' expectations. However, it is possible that these expectations are class centered rather than student centered [57]. Teachers may have expectations for their entire class and classroom characteristics may predict these expectations [58]. Furthermore, the way that teacher background characteristics predict their expectations, remains understudied. Do teachers base their expectations on students' background characteristics or do their own views and experience also matter? Future research is important to assess these findings furthermore.

A third limitation is that the teacher expectation literature contains no agreed-on items for measuring expectations. This possibly follows from the fact that expectations are not uniquely defined. In an attempt to overcome this, we have included items that have been used by various authors before. Although we found the items in our study to probe a single underlying construct (Cronbach's α = .83), the lack of an unique operationalization for teachers' expectations hinder the generalizability and comparability with other studies in the domain.

A final limitation of the study concerns the timing of the measurement of teachers' expectations. The expectations were not measured at the beginning but in the middle of the academic year (February 2003). Therefore, the perceptions of the teacher could already be formed by daily interactions with the children. However, as teachers use prior achievement to form their expectations, any interaction between the teacher and the child may lead to an underestimate of the effect of teachers' expectations. This because the model attributes some of the effect of expectations on later achievement to prior achievement.

### Conclusion

Taken together, the findings in this study suggest that teachers play a role in enlarging existing socio-economic background differences between children, with differential effects for majority and minority children.
